# The Geriatric Virtual Escape Room in Pharmacy Education: Female Students Escape Significantly Faster than Male Students

**DOI:** 10.3390/pharmacy10020036

**Published:** 2022-03-08

**Authors:** Aisha F. Badr

**Affiliations:** Pharmacy Practice Department, King Abdulaziz University Faculty of Pharmacy, Jeddah 22252, Saudi Arabia; afbadr@kau.edu.sa; Tel.: +966-555-660-722

**Keywords:** geriatrics, pharmacy education, escape room, gamification, educational game, virtual teaching

## Abstract

Due to COVID-19 and the limitation of face-face teaching, electronic adaptation for formative and continuous assessment methods were greatly used and documented between 2020 and 2021. This study aims to implement a virtual escape room that will help assist and refine problem-solving skills in fifth-year pharmacy students by reviewing Beer’s criteria and selecting the most appropriate management. A descriptive cross-sectional study was conducted following the implementation of the virtual escape room using google form. Students had to unlock five puzzles using Beer’s criteria. To evaluate pharmacy students’ perception of this method, they completed a survey to identify their views of the game. Of the 128 students enrolled in the geriatric course, all were able to escape (100%). A one-sample t-test indicated statistical significance between gender. Female students escaped statistically faster than male students (*p* < 0.00002) and were more likely to recommend the game to other students and thought the game encouraged them to think of the material in a new way, whereas male students were more neutral towards it. In conclusion, the geriatric virtual escape room was successfully implemented as a pilot innovative method to assist in virtual learning. However, future studies should investigate virtual gamification in pharmacy education and its impact on learning, as well as identify if there were any gender-specific differences in using these tools.

## 1. Introduction

Gamification in pharmacy education showed great impact in accentuating student learning and engagement and is greatly supported by the American Association of Colleges of Pharmacy (AACP) Academic Affairs Committee 2013–2014. The report supports the use and development of serious games to enhance both pharmacy and interprofessional education and further encourage colleges and pharmacy schools to use serious games for learning and professional development [1].

Gamification, which is also known as “educational games”, “serious gaming”, and “Game-Based-Learning”, is when typical elements of a game are applied in non-game areas of academic activities in an attempt to promote student knowledge, interest, and engagement [1,2]. A sense of “unforced learning” that is perceived by students is reported to make the learning process more fun and enjoyable [3].

Escape rooms are one example of gamification in pharmacy. These are typically known as a live-action team-based mission made of multiple puzzles that need to be unlocked during a specific allotted time in order to “escape”. Many published papers described the efficacy of such a model, as well as an increase in knowledge and improvement in clinical skills. Moreover, students have perceived pharmacy educational escape rooms to positively impact their engagement, clinical thinking, problem-solving, teamwork and application of learned in-class concepts [4,5,6,7,8,9,10], skills that are all needed in pharmacy real-world and day-to-day tasks. 

Due to COVID-19 and the limitation of face-face teaching, electronic adaptation for formative and continuous assessment methods were greatly used, encouraged and heavily documented internationally between 2020 and 2021. Some used Kahoot, google forms, and other faculty/university applications and electronic blackboard assessment tools [11,12,13,14,15]. The challenge was to adapt such a method in a virtual way that can still have students work as a group and have interactive, fun, and variable puzzles to unlock, hence the development of virtual escape rooms (VERs) [16]. 

There are a couple of studies that described their experience and adaptation of virtual interactive learning during COVID-19. Most of these studies found a similar perception of VERs to previously documented perceptions of live-escape rooms [17,18,19,20,21,22]. However, none of these virtual escape rooms covered pharmacotherapy courses. 

King Abdulaziz University Faculty of Pharmacy separates both male and female students; however, both are given the same handouts, references, and lectures. Although lectures are given by two different faculty members, both are ambulatory care clinical pharmacists who received the same ASHP-accredited training with a geriatric focus.

The geriatric virtual escape room is an innovative solution, developed to engage students in a virtual group activity and adapt to challenges faced with virtual learning, as well as enhance students’ research, teamwork, and problem-solving skills.

The primary objective of this study is to implement a virtual educational game that will help assist and refine these skills in fifth-year pharmacy students by specifically reviewing Beer’s criteria and selecting the most appropriate management accordingly. The secondary objective of the study is to evaluate the perceived value of this method between both male and female students and assess any differences between them. 

## 2. Materials and Methods

A two-week geriatric course (10 + 2 contact hours) was followed by the implementation of an innovative geriatric-virtual escape room (G-VER) using google form^®^. The aim specifically targeted the student learning outcomes of this course, which include the following:

- Review and identify potentially inappropriate medications (PIMs) using Beer’s criteria.
- Identify reasons to avoid certain medications in older adults.
- Recommend safer alternatives in certain disease states using Beer’s criteria.
- Conduct a simple literature review using the internet to identify changes made by the 2019 Beer’s criteria.
- Identify active ingredients in OTC brand names that may contribute to potential risks in the elderly.

A total of 128 P5 PharmD students (71 female and 57 male) were gathered online via Zoom^®^ before sending the google form^®^ link of the G-VER. The zoom meeting explained (1) the required time to complete the task (90 min), (2) the G-VER plot/concept, explained below, (3) the importance of reviewing Beer’s criteria [23], and (4) group division of 4–5 students max per group.

To gain the allotted 2 marks of this assessment, students were required to participate regardless of escaping the G-VER or not. The form collected their names and university computer ID number for grading purposes only.

The game was designed by an assistant professor teaching the course and was validated by 3 other assistant professors for difficulty, appropriateness, and time needed to complete each puzzle. The time spent creating the VER google form plot, puzzles, and pictures was approximately 5–6 h.

### 2.1. G-VER Concept and Plot

**Figure 1:**
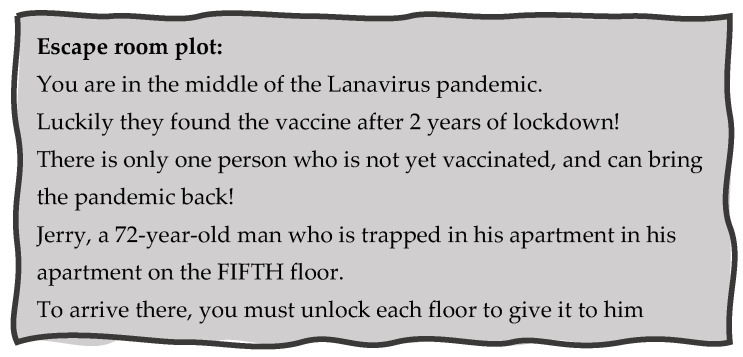
G-VER escape room plot.

The escape room plot the students to get “Jerry”, a 72-year-old man who is trapped on the 5th floor, vaccinated during a made-up pandemic called “Lanavirus”. (Figure 1) To reach Jerry, students had to work in groups to unlock each floor’s puzzle, using Beer’s criteria as a guide. Each floor unlocked the other and so on.

There was a total of five complex puzzles focused on potentially inappropriate medications that are listed on Beer’s criteria. Each puzzle was designed in a way to cover the different student learning outcomes of the geriatric course (mentioned above) and to elicit student engagement and research skills (Table 1). All groups were allowed two hints for any of the puzzles, and they all had Beer’s criteria as a reference. Communication between student groups and the faculty member was conducted via zoom during the allotted time of the activity.

### 2.2. G-VER Perception

Following IRB approval from King Abdulaziz University Faculty of Pharmacy (PH-1443-04), a descriptive cross-sectional study was conducted to evaluate pharmacy student perception of the virtual educational method used. After students completed the virtual escape room, an anonymous survey link, using a google form, was sent to all students and asked for their voluntary participation in the G-VER perception. 

The survey was adapted from a diabetes-themed escape room [5]. There was a total of 10 questions, with a perception scale of a five-point Likert scale ranging from “1 = strongly disagree” to “5 = strongly agree”.

## 3. Results

Of the 128 students enrolled in the geriatric course, all were able to escape the room in the allotted time of 90 min (100%). The average time to complete the G-VER was statistically faster in female students (M = 59, SD = 8.31) compared to male students (M = 65, SD = 9.06) *p* = 0.00002. The fastest group to escape the room was a group of female students who were able to escape in only 41 min. One hundred and twenty-two students (68 females and 42 males) completed the VER voluntary perception survey (95.31% response rate) (Table 2). Overall, a great difference in the G-VER perception was perceived between male and female students.

A one-sample *t*-test indicated statistical significance between gender, where females were more likely to recommend the game to other students (*p* = 0.002) and thought the game encouraged them to think of the material in a new way (*p* < 0.001), whereas male students were more neutral towards it.

Furthermore, females thought the game helped them more in assisting and reviewing Beer’s criteria compared to male students (*p* = 0.002 and *p* = 0.007), respectively. Female students were notably more comfortable working in groups virtually compared to male students (*p* ≤ 0.001).

**Figure 2:**
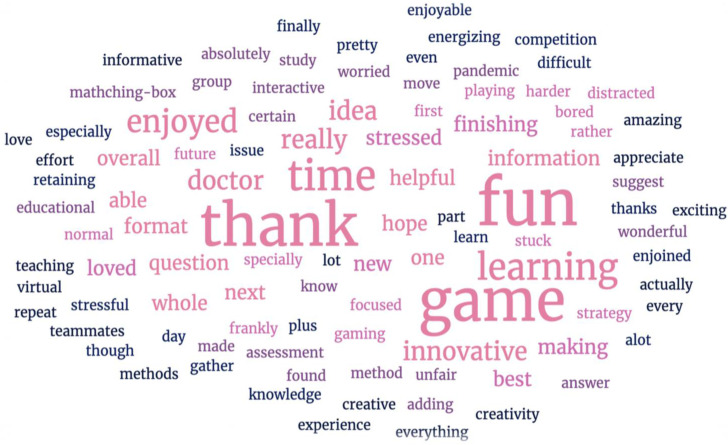
Student feedback presented as a word cloud.

**Figure 3:**
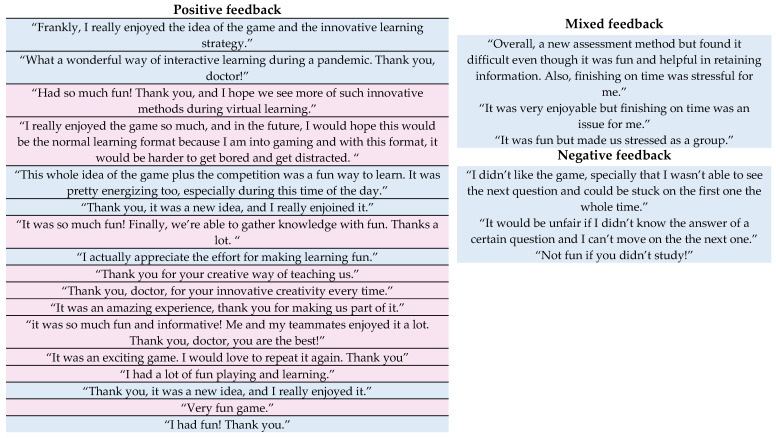
Student feedback and comments on the G-VER. Male feedback is shaded in blue, whereas female feedback is shaded in pink.

There was a total of 23 documented feedback on the game (18.85%). Seventeen positive, three mixed, and three negative feedbacks. A generated word cloud of student feedback on the G-VER revealed 98 words. The most frequented words were fun (10.2%), game (9.18%), and thank you (9.18%) (Figure 2). Positive feedback on the game was mainly appreciative of the creativity, fun, and gaming experience as a whole, as well as wanting more of such educational methods. Mixed and negative feedback was reported by male students who described being stressed, worried about not finishing on time, and not being able to move to the next question (Figure 3).

## 4. Discussion

The 2019 ACCP pharmacotherapy toolkit guides pharmacy schools internationally in developing, maintaining, and modifying the curriculum according to continuous reviewing of medical literature, disease frequency, and the pharmacist’s role in managing certain disease states. Geriatrics is classified as tier one, meaning that students should receive education and training to prepare them to provide collaborative patient-centered care on graduation and licensure [24,25].

Pharmacists are known to play a vital role in medication use in older adults, identifying PIMs, recommending safer alternatives, reviewing drug–drug interactions, and de-prescribing and dose de-escalation [26,27]. The use of such innovative methods of gamification in pharmacy can assist greatly in learning, as it provokes both engagement and a sense of challenge. “Serious games” and their impact is limited in the literature, and escape rooms have shown to be of great impact in healthcare professions such as nursing, medicine, pharmacy, and dentistry [5,16,28,29,30].

Overall, the G-VER design and take of an innovative way to engage students in a virtual group activity during the COVID-19 lockdown challenge were successful, given a 100% success rate of escaping. Students were able to work remotely as a group and were able to meet the assessment method teaching goals as well as student learning outcomes of the geriatric course by completing each puzzle of the game and escaping on time.

This study is the first to report a notable and statistically significant perception of the use of this method between both male and female students. Females were more likely to recommend the game to other students and thought the game encouraged them to think of the material in a new way. The game also assisted females in reviewing Beer’s criteria compared to male students. Moreover, female students were notably more comfortable working in groups virtually compared to male students. Further guidance and understanding of the reasons behind these differences can help aid in selecting a more appropriate game/puzzle to assist in learning.

Although there was no perceived statistical significance, the results do suggest the great impact of virtual escape rooms as an assessment method. Even though there were only 23 reported voluntary feedback (18.9%), most were positive. However, two students felt the need to emphasize two major components; one which was already mentioned in the perception survey, which was (1) stress, and (2) finishing on time. Both were raised by male students. Future studies should look further into the relationship of stress in serious gaming and assess them for qualitative purposes.

The use of voluntary feedback is highly recommended in assessing student perception, and future studies should use open-ended questions to gather any unanticipated questions. This process is beneficial for the future development of any serious game.

Finally, the use of escape rooms in pharmacy is suitable when students apply their critical thinking in order to unlock the puzzles by reviewing guidelines and study materials. Solving each puzzle aims to reinforce concepts learned in class and are anticipated in real-world application. Using the right plot to mimic real-life scenarios can design an effective, reproducible assessment tool that is also fun and engaging to students.

This study had the limitation of not assessing pre and post knowledge, as well as having two different faculty members: one teaching male students and the other teaching female students. Although the material is the same, teaching methods may have been different. Virtual escape rooms could be adapted to many different topics and is a fun, engaging, and innovative way to gain students’ interest and develop research methods. Feedback was very overwhelming, particularly during the severely affected virtual teaching. Using this method is of zero cost and can be modified as necessary every year. Based on the results of this study and student concerns mentioned in the feedback, extending the time limit to 120 min can limit stress related to time constriction. A live escape room is planned for next year’s course, and we will make these changes accordingly.

## 5. Conclusions

The geriatric virtual escape room (VER) was successfully implemented as a pilot innovative method to assist in virtual learning. All methods used during the COVID-19 lockdown should be documented to aid other schools during this ongoing pandemic. This method was fun, easy, and of zero cost to create. Strongly suggest this method as a summative and/or formative assessment. Future studies should look into virtual gamification in pharmacy education and its impact on learning, as well as identify if there were any gender-specific differences in using these tools.

## Figures and Tables

**Table 1:** Geriatric virtual escape room puzzles.

	Educational Objective	Question(s) Asked	Gaming Task
First puzzle	Familiarize students with the escape room	What is Jerry’s name in Cypher code CODE: Cypher code in numbers	Cypher code
Second puzzle	Review and Identify potentially inappropriate medications (PIMs) using Beer’s criteria	Identify (PIMs) from Jerry’s medication list. CODE: First letter sequence of PIMs (ex: AABBCC)	Data hunt
Third puzzle	Identify reasons to avoid certain medications in older adults and provide a safer alternative for each	What is the exception of BZD use in older adults What is digoxin’s maximum dose in the elderly? Why is glipizide considered the safest SU in the elderly? What risk is associated with PPI use in the elderly? CODE: Correct answer sequence (ex: ABCC)	Multiple-choice questions
Fourth puzzle	Conduct a simple literature review using the internet to identify changes made by the 2019 Beer’s criteria	Aspirin was recently added to the 2019 Beer’s criteria for primary prevention due to bleeding risk. What was the name of the trial addressing this risk? CODE: Trial name (ex: LEADER)	Literature review Data hunt
Fifth puzzle	Identify active ingredients in OTC brand names that may contribute to potential risks in the elderly	What active ingredient in Tylenol PM makes it inappropriate to use in the elderly and may cause delirium and increased fall risk? CODE: write the first four letters of the active ingredient	Data hunt

**Table 2:** Geriatric virtual escape room perception scale (N = 122; 68 females (F) and 42 males (M)); “1 = strongly disagree” to “5 = strongly agree”.

Question	Mean (SD)		Strongly Agree (%)		Agree (%)		Neutral (%)		Disagree (%)		Strongly Disagree (%)		*p*-Value
	M	F	M	F	M	F	M	F	M	F	M	F	
I would recommend this activity to other students	3.1 (1.5)	4 (1.3)	28.6	51.5	9.5	16.2	26.2	19.1	14.3	4.4	21.4	8.8	0.002
2. The escape room encouraged me to think about the material in a new way	3 (1.3)	4 (1.3)	19	51.5	14.3	13.2	33.3	22.1	16.7	7.4	16.7	5.9	<0.001
3. The escape room was an effective way to REVIEW Beer’s criteria	3.2 (1.3)	3.9 (1.3)	21.4	47.1	19	19.1	31	17.6	14.3	8.8	14.3	7.4	0.007
4. The escape room was an effective way to LEARN new information related to geriatrics	3.3 (1.3)	3.8 (1.3)	26.2	41.2	14.3	20.6	33.3	22.1	19	8.8	7.1	7.4	0.053
5. The escape room was an effective way to ASSIST my learning of Beer’s criteria	3.1 (1.3)	3.9 (1.2)	19	44.1	16.7	20.6	33.3	20.6	19	10.3	11.9	4.4	0.002
6. I learn better in a game format than in a didactic lecture	3.4 (1.4)	3.4 (1.4)	33.3	32.4	9.5	14.7	35.7	27.9	7.1	13.2	14.3	11.8	1
7. I feel I was able to engage with my teammates virtually	3.2 (1.3)	4.1 (1.3)	23.8	55.9	16.7	19.1	33.3	11.8	11.9	4.4	14.3	8.8	<0.001
8. It was difficult for me to focus on learning because I was feeling stressed or overwhelmed	3.4 (1.3)	3 (1.4)	26.2	17.6	21.4	17.6	26.2	30.9	16.7	10.3	9.5	23.5	0.131
9. The non-educational portions (ex: cipher code) distracted me from learning about Beer’s criteria	2.9 (1.3)	2.7 (1.4)	14.3	14.7	16.7	14.7	35.7	22.1	11.9	19.1	21.4	29.4	0.449
10. In general, I enjoy playing games (video games, board games, social media games, etc.)	3.5 (1.6)	4 (1.3)	40.5	52.9	14.3	19.1	16.7	13.2	9.5	5.9	19	8.8	0.092

## Data Availability

No new data were created or analyzed in this study. Data sharing is not applicable to this article.
